# Supplementary figures and images for: C-type natriuretic peptide facilitates autonomic Ca2+ entry in growth plate chondrocytes for stimulating bone growth
Source: eLife. 2022 Mar 15;11:e71931. doi: 10.7554/eLife.71931 (PMC8923661; doi:10.7554/eLife.71931)

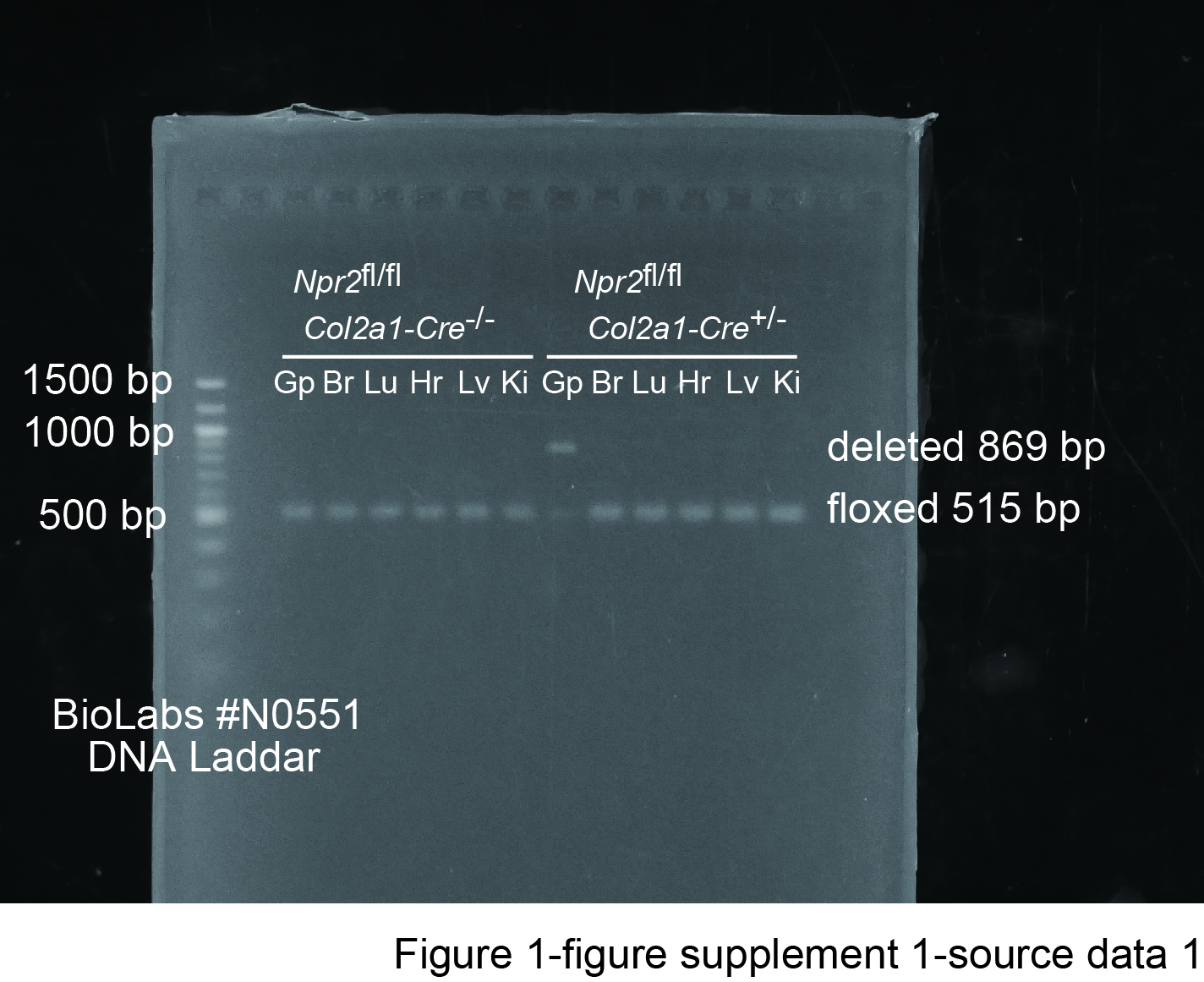

Supplement: Figure 1—figure supplement 1—source data 1. [file elife-71931-fig1-figsupp1-data1.zip › Figure 1-figure supplement 1-source data 1/Figure 1-figure supplement 1-source data 1.jpg]

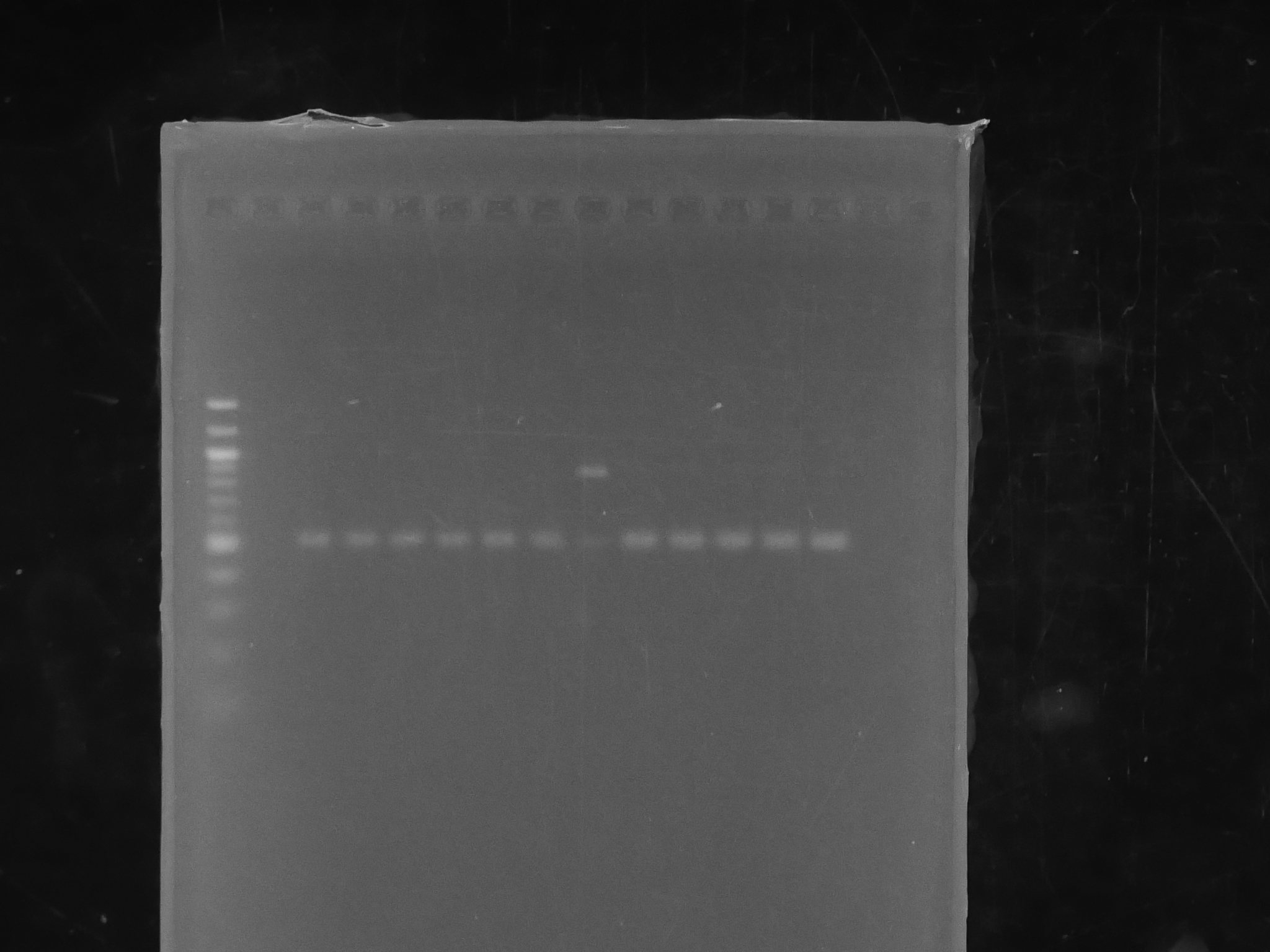

Supplement: Figure 1—figure supplement 1—source data 1. [file elife-71931-fig1-figsupp1-data1.zip › Figure 1-figure supplement 1-source data 1/Figure 1-figure supplement 1-source data 1-unedited.JPG]

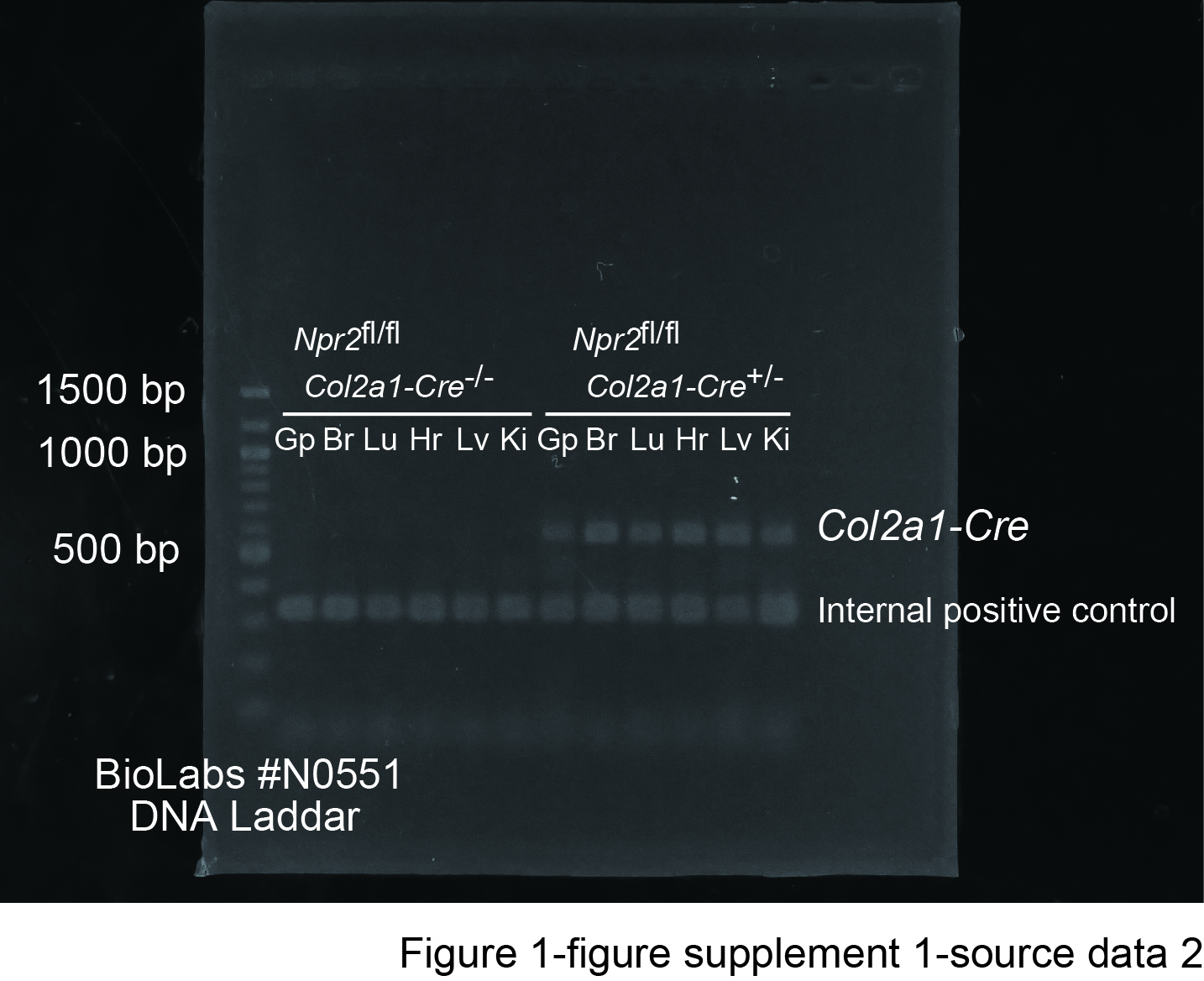

Supplement: Figure 1—figure supplement 1—source data 2. [file elife-71931-fig1-figsupp1-data2.zip › Figure 1-figure supplement 1-source data 2/Figure 1-figure supplement 1-source data 2.jpg]

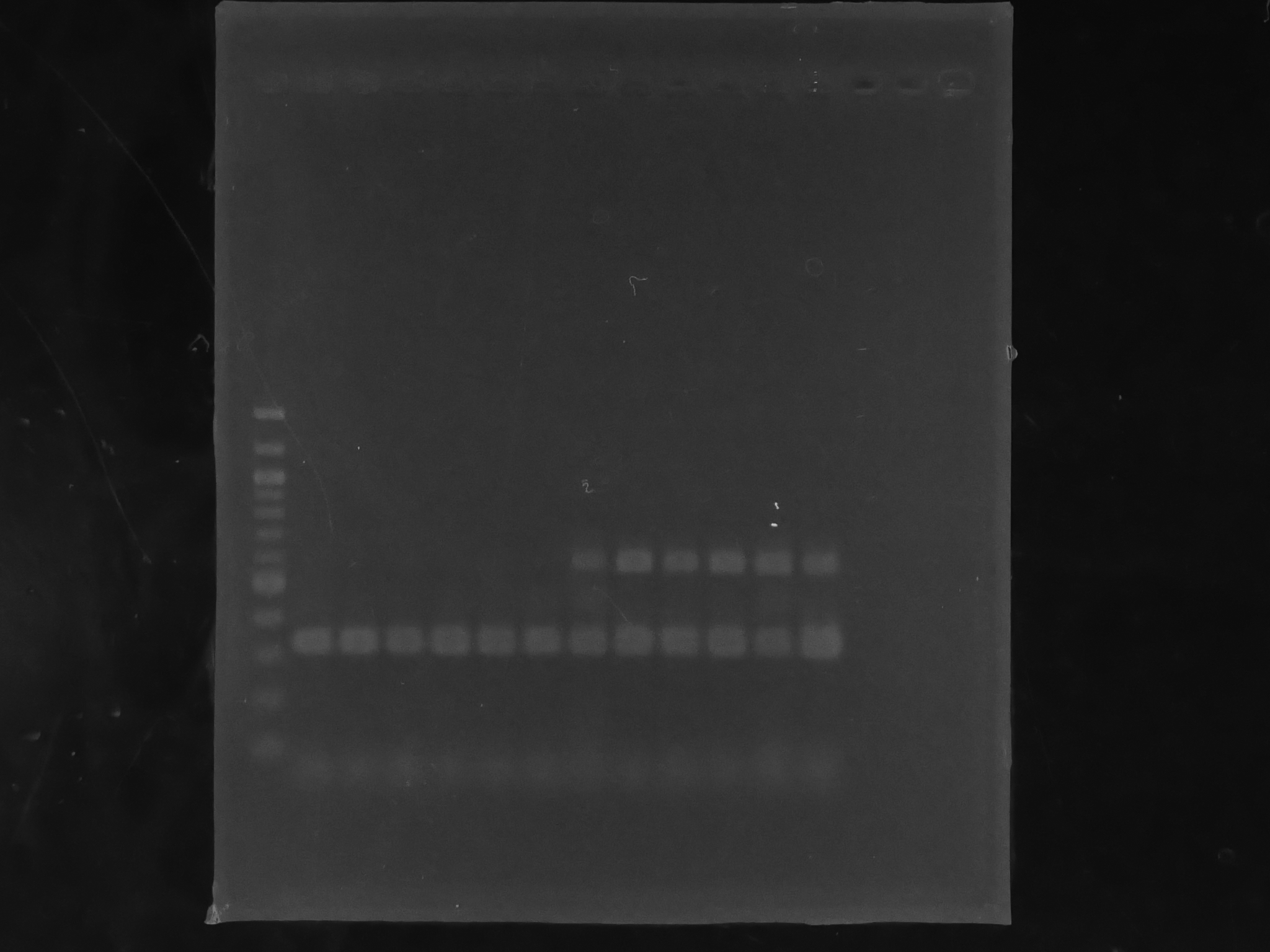

Supplement: Figure 1—figure supplement 1—source data 2. [file elife-71931-fig1-figsupp1-data2.zip › Figure 1-figure supplement 1-source data 2/Figure 1-figure supplement 1-source data 2-unedited.JPG]

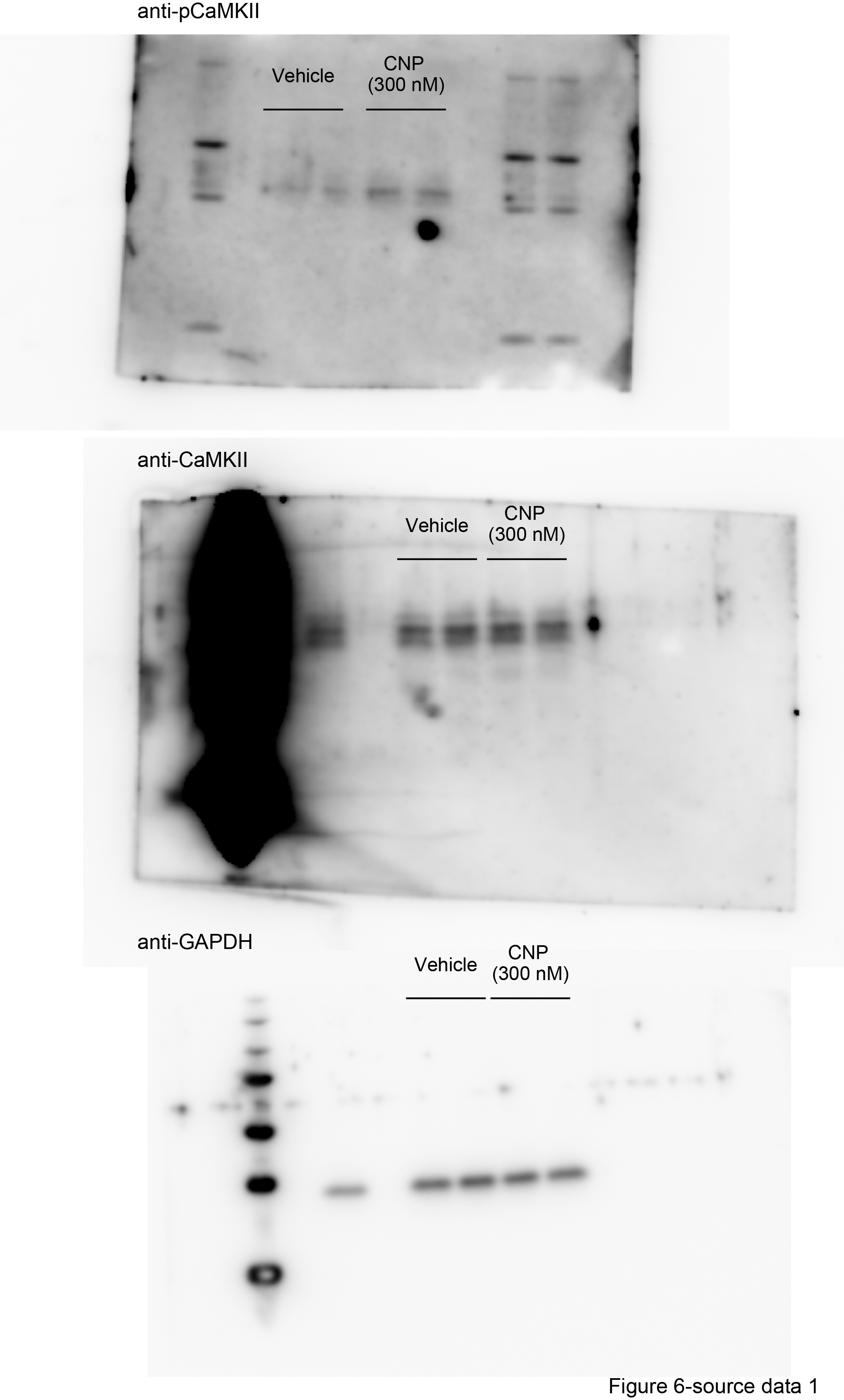

Supplement: Figure 6—source data 1. [file elife-71931-fig6-data1.zip › Figure 6-source data 1/Figure 6-source data 1.jpg]

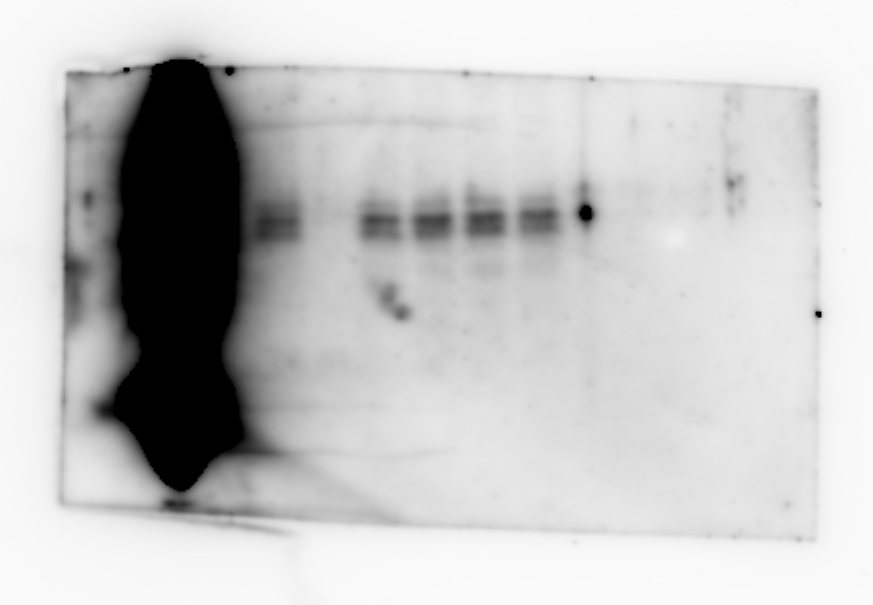

Supplement: Figure 6—source data 1. [file elife-71931-fig6-data1.zip › Figure 6-source data 1/Figure 6-source data 1-CaMKII-unedited.tif]

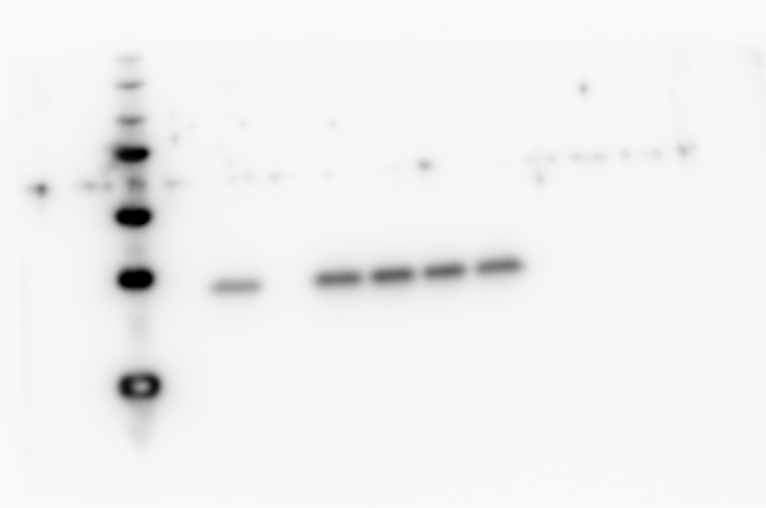

Supplement: Figure 6—source data 1. [file elife-71931-fig6-data1.zip › Figure 6-source data 1/Figure 6-source data 1-GAPDH-unedited.tif]

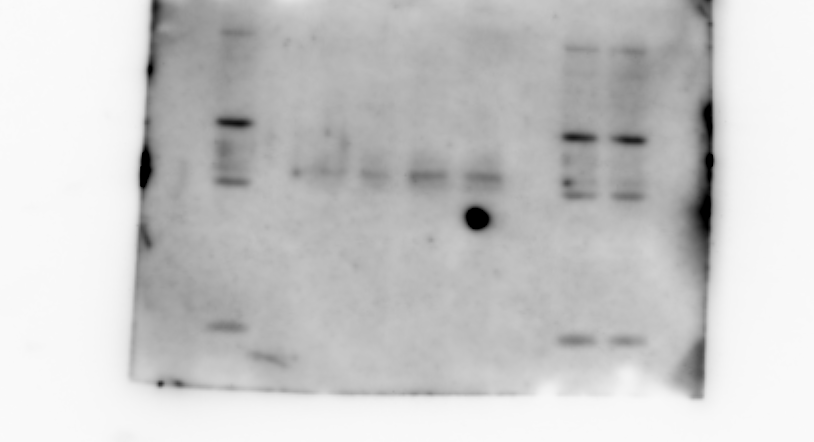

Supplement: Figure 6—source data 1. [file elife-71931-fig6-data1.zip › Figure 6-source data 1/Figure 6-source data 1-pCaMKII-unedited.tif]
